# Socially Accountable Global Health Education Amidst Political Uncertainty and Reactionary Nationalism: A Value Proposition and Recommendations for Action

**DOI:** 10.5334/aogh.2569

**Published:** 2019-09-06

**Authors:** Michael J. Peluso, Marilyn A. DeLuca, Lorenzo Dagna, Bishan Garg, Janet P. Hafler, Elsie Kiguli-Malwadde, Harriet Mayanja-Kizza, Moira A. Maley, Robert M. Rohrbaugh

**Affiliations:** 1 University of California, San Francisco, US; 2 New York University, School of Medicine and Rory Myers College of Nursing, New York, US; 3 Global Health-Health Systems-Philanthropy, New York, US; 4 Vita-Salute San Raffaele University, Milan, IT; 5 IRCCS San Raffaele Scientific Institute, Milan, IT; 6 Mahatma Gandhi Institute of Medical Sciences, Sewagram, Wardha, IN; 7 Yale University School of Medicine, New Haven, US; 8 African Centre for Global Health and Social Transformation (ACHEST), Kampala, UG; 9 Makerere University, Kampala, UG; 10 The University of Western Australia, Perth, AU

Global health (GH) education (GHE) faces an unprecedented threat. A shift in the global political landscape has altered the foundation of globalism upon which medical institutions have built GHE programs. While skeptics may argue that this threat has not yet resulted in challenges to markers of viability like program funding, GH educators must anticipate factors that could threaten the stability and perceived value of their programs in both obvious and insidious ways.

## The era of globalism in which GHE programs developed has ended

Modern GHE programs were developed against a backdrop of globalism. Medical institutions have increasingly encouraged trainees to experience clinical practice in different demographic, socio-economic, and cultural communities; in fact, trainees in high-income countries (HICs) have come to expect these opportunities [1,2,3]. Unprecedented investment by governmental, philanthropic, and academic institutions has decreased the burden of infectious disease, focused attention on non-communicable diseases, supported health service delivery, and advanced the training of physicians and nurses, especially in low- and middle-income countries (LMICs). GHE—defined as *clinical health sciences curricula and experiential learning focused on social accountability and rooted in the concepts of equity, cultural sensitivity, and collaborative, interdisciplinary practice of patient- and population-centered healthcare* [4]—thrived against this backdrop. An increasingly integrated global economy reinforced cross-national political relationships and the expansion of institutional partnerships supporting GHE.

Student training experiences in “away” settings, outside of the context of the home institution, initially took the form of institutions in HICs offering opportunities to complete electives in LMICs. Over time, unidirectional programs gave way to bidirectional opportunities [5,6,7], and institutions in LMICs expanded opportunities in “away” settings within their own countries, other LMICs, and HICs. Evidence showed that students from LMICs experience GHE in similar ways to students from HICs, despite their different frames of origin [8]. As a result, GHE increasingly became defined by experiences in “resource-different” settings [4]. For some participants, GHE led to the pursuit of careers involving the principles underlying GH [9].

GHE also plays a role in the health workforce pipeline. Recent agreements, including the Sustainable Development Goals (SDGs) and Global Strategy on Human Resources for Health: Workforce 2030, incentivized governments to develop the health workforce, strengthen health systems, and support equitable health services. Bidirectional GHE programs contributed to these goals by creating opportunities for educational exchange and training experiences [5,8,10].

## The foundations upon which GHE programs were built are threatened

Following the 2008 recession, weakened national economies began to fuel the rise of populist movements around the world [11]. Several countries began withdrawing from international commitments, reducing development aid, limiting funding for GH programs, and closing their doors to foreign-born trainees and immigrants [12,13,14].

**Figure 1:**
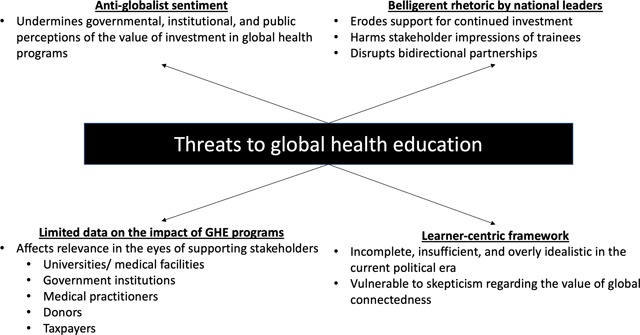
**Threats faced by global health education programs.** This schematic reviews notable threats facing global health education programs in the current political era.

The current political backdrop contrasts with the globalist context that characterized the earlier period of GHE expansion; if it persists, it could threaten GHE programs in several ways (Figure 1).

Individual practitioners who have made a career in GHE are unlikely to waver in their perception of the value of these programs, but a large-scale erosion of support for the principles that underlie GH may influence stakeholders on both sides of GHE partnerships. An eventual outcome might be to undermine financial, administrative, and philosophical investment in these programs, force faculty, administrators, and trainees to reconsider the viability of GHE as a career pathway, and cause interest in participation among trainees to wane. As the quality of downsized programs deteriorates, trainees could view GHE as less worthwhile, initiating a vicious cycle jeopardizing the field.

The rise of nationalism also undermines the ease of and interest in foreign study in direct (e.g., immigration restrictions) and indirect (e.g., devaluation of outsiders’ contributions) ways. If unchecked, this could threaten progress in developing the healthcare workforce, damage exchange programs, and impact the training of healthcare workers in LMICs.

In this current environment, it is essential to demonstrate the value and relevance of GHE. It is equally important to understand the potential shortcomings of the content and framing of GHE to counter forces that could undermine these programs.

## What is the value of GHE in an era of rising anti-global sentiment?

### GHE prepares trainees to practice across diverse settings

GH trainees gain insight into other models of healthcare that transform their career goals and clinical practice perspective [8,9,15]. As a result, trainees develop a more nuanced understanding of healthcare delivery across settings, which may also sharpen their ability to perceive such differences locally. By challenging trainees’ cognitive schema and providing opportunities to experience vulnerability in controlled settings, GHE programs do not just teach trainees about health worldwide, but have the potential to profoundly impact their values, clinical practice, and interactions within their local healthcare systems [16].

Crucially, this has been shown to be true for all participants [5,7,8,9,10], regardless of whether they are a Kenyan student rotating in the U.S., a Canadian student rotating in China, or a student from Delhi, India rotating in Nagpur, India. It may also be true whether their goal is to work at a nationally funded tuberculosis clinic in London, a community health center in rural Australia, or an academic cancer hospital in Los Angeles [5,7,8,9,10].

### GHE creates a workforce equipped to deal with challenges at “home”

The health-related SDGs set targets for countries to achieve by 2030. Nationalist policies that exclude or marginalize individuals either formally through unequal access or informally through stigma are inequitable and undermine the achievement of these targets. Experiential GHE allows trainees to recognize inequity, provide culturally sensitive care, and work with limited resources. As demographics change and health systems strive to do more with less, populations across income settings will need larger numbers of skilled, competent providers of equitable, culturally appropriate, cost-effective care. Those with GHE training are uniquely suited for this role [17,18].

### GHE trains clinician-advocates

Social accountability and justice underlie the missions of many medical institutions. They are also core principles of GHE. GHE programs foster relationships with international partners, drive advocacy, and mentor trainees to be effective change agents [5,8,9,16,17,19,20]. Trainees observe role models working alongside local and national leaders on an array of issues including program implementation, health determinants, social justice, human rights, and access to equitable care. For example, the first-hand experience of negotiating program funding with a Ministry of Health or for essential therapies with an insurance payer develops advocacy skills regardless of setting. Honing these skills helps to translate learned social accountability into practice.

## How must GHE change to ensure it remains relevant?

### Demonstrate that GHE programs support national health priorities

A recent critique of GH programs [21] suggests that there are three overarching rationales that motivate these initiatives: (1) health security; (2) promoting economic and political development; and (3) achieving equitable access to healthcare as a universal right. The third perspective has typically been the driver for most university-oriented GHE programs, while governments may prioritize security and development. Leveraging security or development aspects of GHE may help justify the expansion of existing programs and can ultimately empower these programs to promote a health equity agenda more effectively. GH educators should consider these different priorities as they further develop curricula and identify ways they can advocate for and advance GHE initiatives that also contribute to national priorities. Examples could include the development of curricular components focused on the management of global epidemics or understanding the impact of GH crises on national security.

### Advance alliances between educators working in various “away” settings

Educators in university communities that focus on health equity compete for scarce resources, often with one group advocating for global initiatives and the other advocating for local investments. The former group might be promoting experiential practice in differently resourced settings internationally. The latter may be focused on underserved communities within the same city as a particular training institution. These educators may be teaching the same fundamental skills, but may not recognize their alignment in different “away” settings. The word “global” alone heightens concerns that local projects will be overlooked.

Cooperation between educators who may see one another as competitors despite closely aligned missions will further advance the goals of GHE. For this reason, GH educators should consider developing partnerships with colleagues working in local underserved communities. Some have done this by focusing attention on experiences for trainees in both local and international “away” settings [20]. Educators should be more willing to accept local “away” experiences as GH, develop equitable relationships with local partners, and encourage participation by trainees in these programs under the rubric of GH.

### Acknowledge that “GH” can be practiced under different names—or no name at all

Educators should reject the argument that is sometimes used to disparage GHE—that it is “just ‘health’ in my setting.” This argument is based on the concept that some individuals may practice in “home” settings where GHE *per se* may not be a part of the curriculum, but GH principles are integrated into medical care through an increased focus on population and public health. Colleagues in settings where there is a focus on community medicine and social accountability programs addressing population health that capture the core principles of GHE have much to teach GH educators from different settings [22]. By identifying programs that include the principles of GH, they can demonstrate the universality of these principles, build alliances across settings, and expand the reach of GHE.

### Demonstrate the value of bilateral partnerships and equity in access to GHE experiences

The threat posed by “home” country skepticism regarding experiences “away” is equaled by the risk of “away” site skepticism toward visiting trainees. It is crucial that GHE programs promote reciprocity and, ideally, bidirectionality [5,6,7,10]. The prevalence of such programs is unknown. As evidence that individuals from LMICs engaged in GHE experience similar benefits as those from HICs grows [8,10], these values should ensure equitable access to GHE experiences. At the institutional level, academic partnerships should attempt to demonstrate concrete outcomes like transfers of clinical knowledge and education activities that benefit both partners [7].

### Make GHE curricula relevant to trainees committed to a career in GH

Evidence suggests that clinical trainees who wish to pursue careers in GH require a broader skillset than many short-term clinical experiences provide. It is equally critical for programs to develop non-clinical skills (business/management, political/interpersonal skills) in their participants to make them better equipped to join the GH workforce [23]. GH curricula need to dive deeper into social determinants of health to teach trainees how political and economic factors drive health policy, healthcare delivery, and related policies on issues like immigration, violence, and planetary health. In addition, GH educators must teach trainees about the history of globalization and the recent growth of nationalism. Attention should be paid to orienting trainees involved in GHE programs spanning particularly challenging national (e.g., U.S. and China) or regional (e.g., rural vs. urban) divisions so they can notice whether these divides play out in policy, patient care, and their interactions while at the site.

### Demonstrate an impact on professional practice

Assessing learners in GHE programs and evaluating the programs themselves remains a challenge. At minimum, GH educators should ensure that trainees reflect on what they learned in the “away” setting and consider how this might change their practice at “home” [16]. Efforts to determine the optimal way to provide meaningful assessment of GHE are urgently needed.

GH educators must also evaluate the impact of their programs and align this evaluation with the priorities of national health agendas. For example, one of the most promising findings in early evaluations of GHE programs in the U.S. demonstrated that participants in these programs were more likely to enter primary care [15]. Given the shortage of primary care professionals, this demonstrated the value of GHE to meet health workforce needs. It is crucial to understand the impact of these programs on individuals, institutions, and communities longitudinally to support further investment.

## Future directions

**Figure 2:**
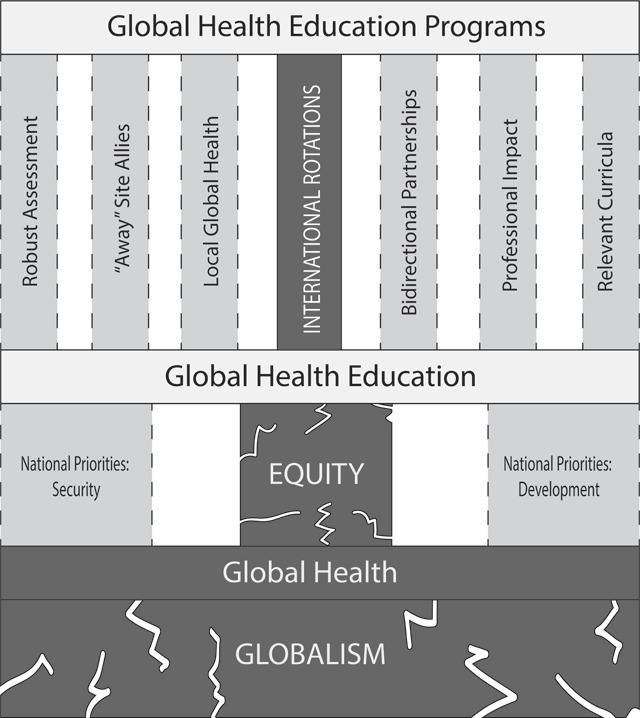
**Schematic of modern global health (GH) education (GHE) programs.** Nationalist political forces potentially damage the foundations (indicated as cracks in the dark gray boxes) upon which GH, GHE, and GHE programs were constructed. The figure summarizes the means by which the structure can be reinforced, as discussed in the manuscript (medium gray boxes).

GHE is at a critical juncture. With concerted efforts of educators and key stakeholders (Table 1, Figure 2), GHE can continue to expand despite growing anti-global political sentiments. Ongoing investment in GHE will develop a workforce prepared for modern threats to global health.

**Table 1:** Strategies for promoting the value of global health education on an anti-global backdrop.


**Frame the constant value of GHE against a backdrop of fluctuating political sentiment**
GHE prepares trainees to practice across diverse settings
GHE prepares professionals for future healthcare challenges
GHE teaches advocacy through effective personal and institutional relationships
**Reframe GHE to emphasize its value despite growing anti-global skepticism**
Demonstrate that GHE programs support global and national health priorities
Advance alliances between global health educators and key stakeholders
Revise curricula to emphasize equity in “home” settings and ensure relevance to careers in GH
Develop robust evaluation to assess influence on professional practice
Integrate health policy and a comparative health systems focus into GHE curricula
